# Effect of dietary stable isotopic ratios of carbon and nitrogen on the extent of their incorporation into tissues of rats

**DOI:** 10.1186/2049-1891-3-14

**Published:** 2012-05-31

**Authors:** Wentao Lv, Tingting Ju, Bing Dong, Boyang Yu, Jingdong Yin

**Affiliations:** 1 State Key Laboratory of Animal Nutrition, China Agricultural University, No.2 West Road Yuanmingyuan, 100193, Beijing, China

**Keywords:** Carbon, Diet, Nitrogen, Stable isotopes

## Abstract

This study was conducted to investigate the effect of different dietary ratios of ^13^ C to ^12^ C or ^15^ N to ^14^ N on their relative incorporation into tissues. Eighty male rats were used in two 21-day feeding trials in which they were fed diets with either high δ^13^C levels (δ^13^C = −13.89‰ and δ^15^N = 2.37‰ in experiment 1 and δ^13^C = −19.34‰ and δ^15^N = 4.73‰ in experiment 2) or low δ^13^C levels (δ^13^C = −17.90‰ and δ^15^N = 3.08‰ in experiment 1 and δ^13^C = −21.76‰ and δ^15^N = 0.53‰ in experiment 2), meanwhile, the dietary δ^15^N levels were designed to two ranks. Blood, liver, adipose and muscle tissues were collected on day 0, 3, 7, 14, and 21 for determination of ^13^ C, ^12^ C, ^15^ N and ^14^ N isotopes. Rat growth rate, antioxidant capacity and metabolic parameters were also assessed. The results indicate that adipose tissue tend to deplete ^13^ C before the stable isotopic ratios achieved final equilibrium. Therefore, feeds with different isotopic signatures had different incorporation rates into tissues. Low dietary ^13^ C levels decreased tissue δ^13^C values whereas high dietary ^13^ C levels did not alter tissue δ^13^C values during the 21-d experiment. Blood δ^15^N values were a reliable parameter in assessing the relative contribution of dietary nitrogen to tissues. This study revealed a relationship between dietary isotopic signatures and their incorporation rates into rat tissues. However, more studies are needed to illustrate the mechanism through which dietary isotopic ratios influence the extent of isotopic incorporation into the tissues.

## Background

Use of stable isotopes has attracted a great deal of interest in physiological and metabolic research as many researchers are unwilling to use radioactive isotopes [1]. The amount of carbon stable isotope ^13^ C varies between the C3 and C4 plants due to their use of different photosynthesis pathways while the abundance of stable nitrogen isotope ^15^ N in plants resembles that of their growing circumstance [2,3]. Since every feedstuff is characterized by its natural stable isotopic signature [4,5], the analysis of stable isotopes in tissues is proposed as a method to evaluate the relative contributions of nutrients from different feed sources to those deposited in tissues [6,7].

Many models have been established to estimate the fractional contribution of various isotope profiles in the diet to those deposited in tissues including one compartment, two compartment, multi-compartment and mixed models [8]. However, the effect of different dietary ratios of ^13^ C to ^12^ C or ^15^ N to ^14^ N on their relative incorporation into tissues remains unknown.

Numerous factors such as diet quality, nutritional status, body size, age, dietary ontogeny, tissue and elemental composition have been reported to affect the extent of deposition of ^13^ C and ^15^ N into tissues [9-12]. The relationship between discrimination factors and dietary isotopic ratios is consistent and dietary isotopic values have been shown to explain 51 % of the variation in isotopic discrimination [13] . In controlled experiments, dietary isotopic values explained 60 % to 98 % of the variation in isotopic discrimination in different tissues of rats [14]. The stable isotopic analysis, which has been used as an important tool for so many years, is conducted to investigate the turnover and deposition of nutrients from the macroscopic view. It is aimed at not a certain nutrient but the relationship between various nutrients. However, few studies have determined the effect of different dietary ratios of ^13^ C to ^12^ C or ^15^ N to ^14^ N on their relative incorporation into tissues. If these discrimination factors are not fully understood, it is impossible to estimate the relative contributions of nutrients by using stable isotopic analysis. Therefore, the present study was carried out to investigate the effect of dietary stable isotopic ratios on their isotopic discrimination in tissues by feeding rats diets providing high or low stable isotopic abundance.

## Methods

### Experimental design and animal model

Sprague–Dawley rats were used as the animal model to do the research. These trials were conducted according to protocols approved by the China Agricultural University Animal Care and Use Committee. Eighty, healthy male, 35 days old rats were used. Every two rats were housed in one cage. The sawdust was laid at the bottom of the cages and replaced with the new and clean sawdust to keep the cages neat every other day. The rats were reared individually on a 12-hour light 12-hour dark cycle and fed commercial feed for a 7 day adaption period before the experiment started. Rats had free access to feed and water. All rats were treated in accordance with the “Guide for Care and Use of Agricultural Animals in Research and Teaching” [15].

Two feeding trials were designed to explore the effects of dietary isotope ratios on stable isotopic incorporation into tissues. Prior to the initiation of the experiment (day 0), four rats were slaughtered in order to determine the initial δ^13^C and δ^15^N values in the tissue of the experimental rats. In the first trial, 38 Sprague–Dawley rats with an average weight of 164.2 ± 2.8 g were randomly divided into two groups (n = 19) and fed one of two diets (Table 1) in which the stable carbon isotopic ratios were designed to be higher than that of the tissues of rats on d 0 (i.e. -19‰ δ^13^C value). The rats in group 1, which was the control group, were fed a diet with a low isotopic ratio (−17.90‰ δ^13^C value and 3.08‰ δ^15^N value) while the rats in group 2 were fed a diet with a high isotopic ratio (−13.89 ‰ δ^13^C value and 2.37‰ δ^15^N value).

**Table 1:** Ingredient and chemical composition of the experimental diets

	Trial 1		Trial 2	
Treatments	1	2	1	2
δ^13^C, ‰	−17.90	−13.89	−21.76	−19.34
δ^15^N, ‰	3.08	2.37	0.53	4.73
Ingredients, %				
Fish meal	0.00	0.00	0.00	20.00
Corn	2.35	44.95	30.85	20.45
Casein	11.00	8.80	0.00	0.00
Soybean meal	0.00	20.00	37.80	0.00
Wheat middling	60.00	0.00	5.00	33.00
Sugar	9.00	9.00	9.00	9.00
Soybean oil	7.00	7.00	7.00	7.00
Cellulose acetate	5.00	5.00	5.00	5.00
Mineral premix	3.50	3.50	3.50	3.50
Vitamin premix^1^	1.00	1.00	1.00	1.00
L-arginine	0.70	0.35	0.00	0.55
L-threonine	0.10	0.00	0.10	0.10
L-methionine	0.03	0.15	0.20	0.03
L-lysine	0.12	0.08	0.30	0.15
Choline	0.25	0.25	0.25	0.25
Nutritional level^2^				
Gross energy, MJ/kg	18.11	18.90	19.19	18.54
Crude protein, %	20.82	20.32	20.78	21.18
Arginine, %	1.80	1.81	1.80	1.83
Histidine, %	0.69	0.69	0.69	0.73
Lysine, %	1.35	1.35	1.39	1.36
Methionine + cysteine, %	0.80	0.82	0.84	0.80
Threonine, %	0.87	0.82	0.87	0.87
Tryptophan, %	0.28	0.25	0.25	0.24
Calcium, %	1.46	1.48	1.48	1.50
Phosphorus, %	1.19	1.11	1.12	1.24

^1^ Including 0.14 % tertiary butylhydroquinone per kg of vitamin premix.

^2^ All nutrient levels were determined.

In the second trial, 38 male rats with an average weight of 163.8 ± 3.3 g were randomly divided into two groups (n = 19) and fed diets in which the stable carbon isotope ratios were formulated to be lower than that of the tissues of rats (i.e. -19‰ δ^13^C value) with the exception of adipose tissue. Rats in group 1 were fed a diet in which the carbon and nitrogen isotopic values were −21.76‰ and 0.53‰, respectively. Rats in group 2 were fed a diet with a carbon isotopic value of −19.34‰ and the nitrogen isotopic value of 4.73‰ (Table 1). The rats were fed the experimental diets for 21 days and rat weight and feed intake were determined weekly to calculate average daily gain and average daily feed intake. Additionally, plasma parameters were determined to examine the influence of dietary stable isotopic ratio on rat metabolic physiology.

The experimental diets were formulated to be isocaloric (18.7 ± 0.2 MJ/kg) and isonitrogenous (20.78 ± 0.18 % CP) by using different ratios of fish meal, corn, casein, soybean meal and wheat middlings. The stable isotope ratios of these five ingredients were measured prior to the initiation of the study and the proportion of these ingredients in the diets were set in order to obtain the target carbon and nitrogen isotopic ratios. The chemical composition of the experimental diets and their stable carbon and nitrogen isotopic ratios are shown in Table 1.

### Chemical analysis of feed ingredients

The feed ingredients were determined for gross energy by adiabatic oxygen bomb calorimeter (Parr Instruments, Moline, IL), crude protein through Kjeldahl N [16], calcium (procedure 4.8.03, AOAC, 2000) [17] and phosphorus (procedure 3.4.11, AOAC, 2000) [17]. Amino acid in the feed of experiment 1 and 2 were analyzed according to the procedures 4.1.11 of AOAC (2000) [17] and the procedure 998.15 of AOAC (1995) [18].

### Sample collection and preparation

In order to collect tissue samples, 50 mg/kg BW of sodium pentobarbital (Beijing Solarbio Science & Technology Company, Beijing, China) was injected into the abdominal cavity of the rats. The abdominal cavity was opened and 5 mL of blood was obtained from the hepatic artery using 9 mL heparinized tubes (Greiner Vacuette, Monroe, NC). The rats were then killed by cervical dislocation. Samples of liver, muscle and adipose tissue were then obtained.

The blood samples were separated into two parts, one part was stored at −80°C for stable isotope analysis, and the other part was centrifuged at 1,200 × g for 15 min to obtain plasma and was then immediately stored at −20°C until analysis. The liver sample was collected from the left lobe and rinsed briefly in physiological saline (0.9 %, m/v) to diminish blood contamination of the tissue. The adipose tissue on the bilateral paradidymis was collected from the belly. The gastrocnemius on the left leg was removed for use as a muscle sample. One part of the tissues were for the determination of stable isotopes, one was for the analysis of antioxidant and metabolic parameters, and one was for bulk density measurement.

All samples involving feed ingredients, feed and tissues were freeze-dried at −40°C for 48 h (Virtis Genesis-250es; SP Scientific, Stone Ridge, NY). The dried samples were milled into finer particles and then passed through 80 mesh screen. Then, the appropriate quantities were removed to tin capsules and compactly packaged to ensure the samples did not leak from the tin capsules. Finally, the tin capsules were weighed for analysis.

### Stable isotope analysis

Stable isotope values are expressed as the ratio of the heavier element to the lighter element, for example, ^13^ C/^12^ C and ^15^ N/^14^ N, which are denoted as δ^13^C or δ^15^N. The natural stable isotopic abundance of carbon and nitrogen was measured in feed, blood, liver, muscle and adipose tissue using a continuous-flow isotope ratio mass spectrometer (Delta Plus XP; Thermo Finnigan, Scientific Instrument Services, Ringoes, NJ). The isotopic signature is expressed in the δ-notation in parts per thousand (‰) according to an equation where *X* is an element, and *H* and *L* are the heavy and light isotopes, respectively. The equation used was as follows:

(1) $$\delta {\text{ X}}_{\text{H}}=\left[{\left({\text{X}}_{\text{H}}/{\text{X}}_{\text{L}}\right)}_{\text{sample}}/{\left({\text{X}}_{\text{H}}/{\text{X}}_{\text{L}}\right)}_{\text{standard}}-1\right]\times 1000$$

For correction of instrumental drift and determination of the inter-batch variability of analyses, standard materials were tested at the beginning, in the middle and at the end of each run. The standard materials were ^13^ C and ^15^ N labeled glycine (Cambridge Isotope Laboratories, Andover, MA). The glycine had previously been calibrated with Pee Dee Belemnite for carbon and atmospheric nitrogen [19]. The δ^13^C of the glycine was −33.3‰ while the δ^15^N for glycine was 10‰. The precision of each isotopic measurement was 0.02‰ and the repeatability of each sample was smaller than 0.2‰. Besides, the discrimination factor for tissues to diet (Δ^15^N _tissue - diet_) used was determined as follows:

(2) $$\Delta^{15}{\text{N}}_{\text{tissue }–\text{ diet}}=\delta^{15}{\text{N}}_{\text{tissue}}-\delta^{15}{\text{N}}_{\text{diet}}$$

### Bulk density measurement

Tissues were weighed, represented as m (g), and then were immersed into 2 mL of water placed in a 5 mL cylinder. The size of the tissues was determined by the increased volume of the water in the cylinder, represented as V (mL). Bulk density, represented as D (g/mL), was determined as follows:

(3) D=m/V

### Antioxidant capacity and metabolic parameters

Liver samples were immediately homogenized in ice-cold phosphate buffered saline (10 mL/g tissue) with a glass homogenizer and the homogenate was centrifuged for 15 min at 1,200 × g. The supernatant obtained and the plasma were used to assay for total antioxidant capacity (T-AOC), total nitric oxide synthase (TNOS), inducible nitric oxide synthase (iNOS), glutathione peroxidase (GSH-Px), superoxide dismutase (SOD), glutamic-pyruvate transaminase (GPT), glutamic-oxaloacetic transaminase (GOT), urea nitrogen, maleic dialdehyde (MDA) and creatinine. All these enzymes activities were determined using commercial kits (Nanjing Jiancheng Bioengineering Institute, Nanjing City, China) in accordance with the manufacturer’s instructions.

### Statistical analysis

The influence of the dietary treatments on ^13^ C and ^15^ N incorporation, growth, antioxidant capacity as well as metabolic parameters were analyzed using the one-way time-repeated GLM procedures of SAS (8.02; SAS Institute Inc., Cary. NC, USA). The interaction between group and time-repeated was also considered. Differences were considered significant when *P* < 0.05.

## Results

### Performance and organ weights

There were no significant differences in growth rate and feed intake of rats which received the different stable isotopic ratio diets in either experiment 1 or 2 (data was not shown). Weights of kidney and heart were not altered by the dietary treatments and neither were the bulk density of liver, kidney and heart (data was not shown).

### Antioxidant capacity and critical metabolic parameters

Plasma parameters and hepatic indexes were determined to investigate the influence of different stable isotopic ratios on the antioxidant and metabolic status of the body. In trial 1, the activities of plasma GPT, GOT, T-AOC, SOD, GSH-Px, creatinine, TNOS, iNOS, plasma urea nitrogen and MDA did not differ between rats fed the two dietary treatments (data was not shown). Similarly, no differences were observed in the activities of T-AOC, SOD, MDA, iNOS, TNOS, GSH-Px in the liver along with the period of dietary treatment extending. However, in trial 2, the activity of plasma GOT (6.1 IU/L vs. 15.8 IU/L, *P* = 0.05) and plasma urea nitrogen (18.3 mmol/L vs. 31.0 mmol/L, *P* = 0.06) tended to decrease in the rats that ingested the diet with δ^13^C of −21.76‰ and δ^15^N of 0.53‰, whereas plasma MDA tended to increase (13.3 nmol/mL vs. 10.9 nmol/mL, *P* = 0.06) compared with those of rats that ingested δ^13^C of −19.34‰ and δ^15^N of 4.73‰.

### Stable isotope abundance in tissues

Stable carbon isotopic values showed significant tissue-specificity between adipose tissue and liver, muscle, blood; while each tissue, involving liver, blood, and muscle, had their own nitrogen isotope signature. In particular, both in trial 1 and 2, the adipose tissue ^13^ C value was about 3.0‰ lower than that of the other tissues, while ^13^ C values of the liver, blood and muscle were similar. There were observed relationships among ^15^ N values of the different tissues with liver ^15^ N > blood ^15^ N > muscle ^15^ N.

In trial 1 in which rats were fed with higher ^13^ C diets relative to rat tissues, no difference was observed in the δ^13^C values of the liver, muscle and blood between treatments, whereas adipose tissue δ^13^C values tended to be altered by dietary treatment (*P* = 0.05; Table 2). Lipid δ^13^C values significantly decrease as the period of time the rats exposed to the experimental diet increased (*P* < 0.01). In contrast, δ^13^C values of liver, muscle and blood were not altered by dietary δ^13^C values nor the length of the time the rat was exposed to the experimental diets (*P* > 0.05). There was no significant interaction between the dietary δ^13^C value and exposure time on ^13^ C values of the liver, muscle, blood and adipose tissue (*P* > 0.05).

**Table 2:** Influence of diet δ^13^C values (‰) on δ^13^C incorporation into tissues in Trial 1

Tissue	Treatments		Time, d					*P* value			
	δ^13^C = −17.90‰	δ^13^C = −13.89‰	0	3	7	14	21	SEM	Group	Time	Group × Time
	δ^15^N = 3.08‰	δ^15^N = 2.37‰									
Liver	−19.08^a^	−19.45^a^	−18.95^a^	−19.01^a^	−18.98^a^	−20.02^a^	−19.65^a^	0.29	0.39	0.35	0.22
Muscle	−19.51^a^	−19.45^a^	−19.32^a^	−19.51^a^	−19.74^a^	−19.64^a^	−19.17^a^	0.14	0.74	0.23	0.83
Blood	−19.22^a^	−19.10^a^	−19.20^a^	−19.18^a^	−19.09^a^	−19.19^a^	−19.17^a^	0.09	0.33	0.93	0.34
Lipid	−23.16^a^	−22.06^a^	−22.49^a^	−22.25^a^	−22.89^a^	−23.76^b^	−24.16^b^	0.22	0.05	< 0.01	0.20

^a, b, c^ Mean values within a row with unlike superscript letters were significantly different (*P* < 0.05).

The liver δ^15^N value was significantly altered by dietary nitrogen isotopic values (*P* < 0.01), but no change was observed in the muscle between dietary treatments (Table 3). Blood δ^15^N value tended to be altered by dietary δ^15^N values (*P* = 0.06). Meanwhile,the lower ^15^ N diets relative to the initial tissue δ^15^N significantly reduced δ^15^N values of liver and blood along with extending length of time the rats were exposed to the experimental diets (*P* < 0.01). There was no significant interaction between the diet δ^15^N value and the length of time the rats were exposed to the dietary treatment on δ^15^N values of liver, muscle and blood (*P* > 0.05).

**Table 3:** Influence of diet δ^15^N values (‰) on δ^15^N incorporation into tissues in Trial 1

Tissues	Treatments		Time, d					*P* value			
	δ^13^C = −17.90‰	δ^13^C = −13.89‰	0	3	7	14	21	SEM	Group	Time	Group × Time
	δ^15^N = 3.08‰	δ^15^N = 2.37‰									
Liver	8.45^a^	7.76^b^	9.04^a^	7.43^c^	7.72^bc^	7.83^bc^	8.40^ab^	0.19	< 0.01	< 0.01	0.61
Muscle	5.82^a^	5.77^a^	5.85^a^	5.59^a^	5.76^a^	6.30^a^	5.56^a^	0.19	0.80	0.30	0.89
Blood	6.90^a^	5.90^a^	8.26^a^	7.94^a^	7.75^a^	4.84^b^	3.93^b^	0.42	0.06	< 0.01	0.52

^a, b, c^ Mean values within a row with unlike superscript letters were significantly different (*P* < 0.05).

**Figure 1:**
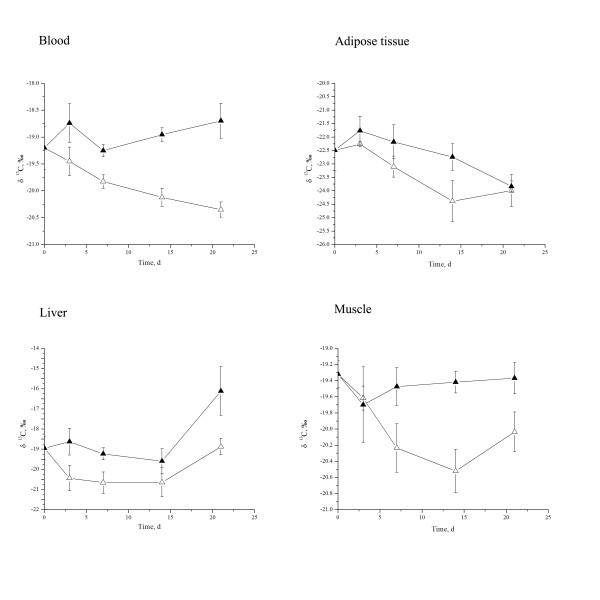
**δ**^**13**^**C value change in Sprague–Dawley rat blood, adipose tissue, liver, and muscle over time.** Open triangles are treatment δ^13^C = −21.76‰, δ^15^N = 0.53‰ and filled triangles are treatment δ^13^C = −19.34‰, δ^15^N = 4.73‰. Data are expressed as mean ± SE.

In trial 2, in which rats were fed with equal (as control group) or lower δ^13^C value (treatment group) relative to that of the tissues except adipose tissue, it is interesting to find that δ^13^C values of the liver (*P* < 0.01), muscle (*P* < 0.01) and blood (*P* < 0.01) were significantly decreased by the diet with δ^13^C of −21.76‰ compared with the other group (Table 4; Figure 1). However, there was no influence of duration of exposure to the treatments on δ^13^C values of the muscle and blood (*P* > 0.05). Dietary of low δ^13^C value tended to decrease the adipose tissue δ^13^C values (*P* = 0.07) while the length of time the rats were exposed to the treatments significantly reduced adipose tissue δ^13^C values (*P* = 0.01). We could not understand the shifting of liver δ^13^C values as it keep constant from d 0 to d14, then increase at d 21, which warrants more meticulously study. There was no interaction between dietary treatment and the duration of treatment on the δ^13^C values of the liver, muscle and adipose tissue, although the interaction tended to increase in the blood δ^13^C value (*P* = 0.07).

**Table 4:** Influence of diet δ^13^C values (‰) on δ^13^C incorporation into tissues in Trial 2

Tissues	Treatments		Time, d					*P* value			
	δ^13^C = −21.76‰	δ^13^C = −19.34‰	0	3	7	14	21	SEM	Group	Time	Group × Time
	δ^15^N = 0.53‰	δ^15^N = 4.73‰									
Liver	−19.78^b^	−18.44^a^	−18.95^ab^	−19.43^b^	−19.87^b^	−20.12^b^	−17.65^a^	0.32	< 0.01	< 0.01	0.31
Muscle	−19.95^b^	−19.46^a^	−19.32^a^	−19.66^ab^	−19.85^ab^	−19.97^a^	−19.70^ab^	0.14	< 0.01	0.20	0.15
Blood	−19.80^b^	−18.92^a^	−19.20^a^	−19.14^a^	−19.54^a^	−19.54^a^	−19.52^a^	0.11	< 0.01	0.15	0.07
Lipid	−23.14^a^	−22.60^a^	−22.49^ab^	−22.01^a^	−22.64^ab^	−23.45^bc^	−23.91^c^	0.28	0.07	< 0.01	0.63

^a, b, c^ Mean values within a row with unlike superscript letters were significantly different (*P* < 0.05).

**Figure 2:**
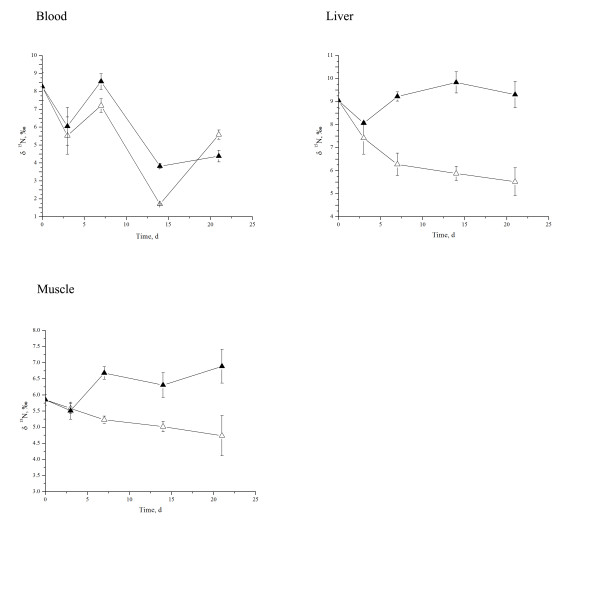
**Nitrogen isotopic value change in Sprague–Dawley rat blood, liver, and muscle over time.** Open triangles are treatment δ^13^C = −21.76‰, δ^15^N = 0.53‰ and filled triangles are treatment δ^13^C = −19.34‰, δ^15^N = 4.73‰. Data are shown as mean ± SE.

In trial 2, δ^15^N values of both liver and muscle were significantly changed by the different dietary δ^15^N values (Table 5; Figure 2). Both liver and blood δ^15^N values decreased along with the increased duration of exposure to the dietary treatments (*P* < 0.01). The interaction between dietary δ^15^N value and the duration of time the rats were exposed to the dietary treatment significantly influenced δ^15^N values of the liver (*P* < 0.01) and muscle (*P =* 0.01).

**Table 5:** Influence of diet δ^15^N values (‰) on δ^15^N incorporation into tissues in Trial 2

Tissues	Treatment		Time, d					*P* value			
	δ^13^C = −21.76‰	δ^13^C = −19.34‰	0	3	7	14	21	SEM	Group	Time	Group × Time
	δ^15^N = 0.53‰	δ^15^N = 4.73‰									
Liver	6.88^b^	8.99^a^	9.04^a^	7.74^b^	7.91^b^	7.85^b^	7.41^b^	0.25	< 0.01	0.02	< 0.01
Muscle	5.24^b^	6.28^a^	5.85^a^	5.55^a^	5.96^a^	5.66^a^	5.81^a^	0.18	< 0.01	0.77	0.01
Blood	5.60^a^	6.01^a^	8.26^a^	5.79^b^	7.87^a^	4.98^b^	3.93^c^	0.35	0.34	< 0.01	0.20

^a, b, c^ Mean values within a row with unlike superscript letters were significantly different (*P* < 0.05)

## Discussion

In the present study, we investigated whether different dietary stable isotope ratios influenced rat growth rate, critical parameters relating to body antioxidant capacity or metabolism. Our results showed that dietary isotopic signature had no or little effect on rat body antioxidant capacity, metabolism or growth rates.

Isotopic ratios in foods consumed are reflected in the tissues, proportionate to the amount assimilated for each ingredient source, after accounting for discrimination against heavier isotopes in the digestion and assimilation process [20,21]. Thus, stable isotope analysis is frequently used to quantify contributions of different food sources to an animal’s diet and nutrient routing [14,22], which requires a priori estimates of discrimination factors. However, the discrimination factors, particularly, the diet-dependent discrimination factors that influence isotopic incorporation into the tissues remain unknown. It has been emphasized that potential sources of variation for discrimination factors should not be overlooked, which underpins the isotope model used in studies of ecology and animal nutritional physiology [13].

Many studies have attempted to determine the abundance of ^13^ C and ^15^ N in animal tissues and these studies have shown that stable isotopic signature varies among tissues [9,23-25]. The reasons for tissues differing in δ^13^C and δ^15^N values were summarized by Martínez del Rio [8]. For δ^13^C values, the adipose tissue content and amino acid composition of tissues are two important candidates. It has been proved that adipose tissue synthesis is accompanied by depletion of ^13^ C [20]. Meanwhile, the difference in δ^15^N value among tissues is due to differences in their amino acid content and the isotopic composition of individual amino acids. However, effect of different levels of dietary stable isotopic ratios relative to the animal tissues on their incorporation into tissues remains unrevealed.

Prior to the initiation of this experiment, when the rats were fed commercial diets, the ranking of tissue isotopic abundance for ^13^ C was liver, muscle and blood > adipose tissue. The same distribution order was maintained when rats ingested different ^13^ C diets for the 21-day experiment. These results were somewhat different from some other reported studies. For example, tissue δ^13^C values were shown to vary among the tissues with the order of abundance being hair > brain > muscle > liver > adipose tissue in the gerbil [23]. There are limited data on the tissue ^13^ C turnover of rats, although the half-life of blood carbon was reported as 24.8 days [26]. We designed the duration of the present trials based on the mouse, using a half-life of liver carbon of 6.4 days [23] and that of muscle of 16.5 days [27]. Since rats have a slower metabolic rate per unit mass than mice [26], the half-life for rat tissue carbon might be longer than that of mice. No difference was observed in the present study for muscle, liver and blood ^13^ C which might be partly due to the fact that these tissues had not equilibrated with dietary ^13^ C during the 21 day experiment. Before isotopic incorporation achieves equilibrium, the metabolic rate that is tissue protein turnover, including synthesis and catabolism, shapes the stable isotopic composition of the bodies as well as the tissues, although the growth, metabolic rate and nutritional status of the animal providing the tissues would also influence it to certain extent [28].

Regardless of diet, adipose tissue δ^13^C value was about 3.0‰ lower than that of other tissues, which was in accordance with previous studies [29]. It has been suggested that adipose tissue depletes ^13^ C faster than other tissues, whereas liver, muscle, blood and hair enrich ^13^ C [8,14,24]. Increases in the mass of tissues through growth have an additional dilution affect which results in faster equilibration to the new diet than would occur by metabolic turnover alone [22].

In the current study, the abundance of tissue ^15^ N values were ranked as liver > blood > muscle. The discrimination factor (δ^15^N _tissue_ - δ^15^N _diet_) varied from 1.14‰ to 5.81‰, which is similar to previous studies [14,30].

Most animal tissues undergo continual incorporation of stable isotopes including ^13^ C and ^15^ N, and so the stable isotope values change over time depending on the diet fed and tissue-specific metabolic rates [31]. In the present study, an interesting phenomenon was observed in that different dietary δ^13^C values, compared with that of the tissues, differentially modulated the stable carbon isotope incorporation into tissues. When rats were fed low stable carbon isotopic ratio diets, δ^13^C values in liver, muscle and blood decreased significantly which is consistent with previous research [26]. However, we found that when rats were fed high isotopic ratio diets, the δ^13^C values of the tissues were not altered during the 21-day experiments. It seems that the tissues tend to assimilate ^12^ C rather than ^13^ C and remain at a certain δ^13^C value. The finding that adipose tissue δ^13^C values were unaltered by dietary stable carbon isotope values in the short term might be due to the fact that adipose tissue has the highest percentage of carbon element among the tissues analyzed.

Isotopic composition turnover rates vary among tissues, with high rate in tissues such as blood and liver, somewhat lower rates in muscle, and low rates in long-lived tissue such as bone [23]. In the present study, it was surprising to find that the liver δ^15^N value was rapidly decreased at day 3 and then remained constant over the remaining 21 days of the dietary treatments. However, we cannot explain why the liver δ^15^N value was decreased at day 3 and then remained the δ^15^N level. Since liver is a central organ of metabolism in the body, perhaps the liver isotopic composition change was balanced by the bodies’ metabolism. Blood δ^15^N value also decreased significantly at day 3 or 14, and further decreased to day 21. The time divergence existing in the two trials might be due to the protein quality of the diets. Because a 21-day dietary treatment is essentially a short-term study, we did not observe a decrease in muscle δ^15^N value during the experiments.

Combining the results of two feed trials, it could be concluded that the liver is the most sensitive organ reflecting the difference of dietary δ^15^N values, the muscle followed second among three tested organs. However, the muscle was resistant to further decrease in δ^15^N once it obtained a new equilibrium of δ^15^N values. Blood δ^15^N value was similar between dietary treatments but it was decreased along with the increased duration of rat exposure to the dietary treatment, which could be accounted for that the difference of δ^15^N between dietary treatments is far smaller than that between the diets and the initial blood δ^15^N. In fact, blood δ^15^N reduced linearly together with the increased length of rat exposure to the lower δ^15^N diets, which suggested that blood δ^15^N values be suitable for assessing the extent of dietary nitrogen contribution to the rat’s tissues. Furthermore, the relationship between dietary isotopic ratios and their incorporation rate in tissues warrant more studies. Particularly, in estimating discrimination factors of stable isotopes, the half-life of the stable isotopes in given tissues should be further researched.

Collectively, dietary stable isotopic ratios of carbon or nitrogen may play a vital role in estimating their incorporation into the tissues in different animals. Dietary ^13^ C incorporation had been shown to be more complex than ^15^ N. In the short term, before the stable isotopes achieved equilibrium, the tissues tend to deplete ^13^ C during the turnover process. Therefore, different levels of dietary δ^13^C values have been shown to have different incorporation rates into the tissue. Lower dietary δ^13^C values decrease the tissue δ^13^C values, whereas the higher dietary δ^13^C values did not alter the tissue δ^13^C values as quickly.

## Abbreviations

T-AOC = Total antioxidant capacity; TNOS = Total nitric oxide synthase; iNOS = Inducible nitric oxide synthase; GSH-Px = Glutathione peroxidase; SOD = Superoxide dismutase; GPT = Glutamic-pyruvate transaminase; GOT = Glutamic-oxaloacetic transaminase; MDA = Maleic dialdehyde.

## Competing interests

The authors declare that they have no competing interests.

## Authors’ contributions

The authors’ responsibilities were as follows: JY designed the study; WL, TJ and BY carried out the animal feeding trials, collected and analyzed the samples; BD provided technical expertise; JY and WL performed statistical analysis, WL wrote the manuscript; JY and BD revised the manuscript; Yin obtained funding. All authors have read and approved the final manuscript. And there is no conflict of interest between authors.

## References

[B1] Gannes LZ del Rio CM Koch P Natural abundance variations in stable isotopes and their potential uses in animal physiological ecology Comp Biochem Phys A 1998 119 725 737 10.1016/S1095-6433(98)01016-2 9683412

[B2] Kelly S Heaton K Hoogewerff J Tracing the geographical origin of food: The application of multi-element and multi-isotope analysis Trends Food Sci Tech 2005 16 555 567 10.1016/j.tifs.2005.08.008

[B3] Ghidini S Ianieri A Zanardi E Conter M Boschetti T Iacumin P Bracchi PG Stable isotopes determination in food authentication: A review Ann Fac Medic Vet Univ Parma 2006 XXVI 193 204

[B4] Boutton TW Tyrrell HF Patterson BW Varga GA Klein PD Carbon kinetics of milk formation in holstein cows in late lactation J Anim Sci 1988 66 2636 2645 3198542 10.2527/jas1988.66102636x

[B5] Gannes LZ O’Brien DM Martínez del Rio C: Stable isotopes in animal ecology: assumptions, caveats, and a call for more laboratory experiments Ecology 1997 78 1271 1276 10.1890/0012-9658(1997)078[1271:SIIAEA]2.0.CO;2

[B6] Post DM Using stable isotopes to estimate trophic position: Models, methods, and assumptions Ecology 2002 83 703 718 10.1890/0012-9658(2002)083[0703:USITET]2.0.CO;2

[B7] Bujko J Schreurs VVAM Nolles JA Verreijen AM Koopmanschap RE Verstegen MWA Application of a [13CO2] breath test to study short-term amino acid catabolism during the postprandial phase of a meal Brit J Nutr 2007 97 891 897 10.1017/S0007114507433049 17381966

[B8] Martínez del Rio C Wolf N Carleton SA Gannes LZ Isotopic ecology ten years after a call for more laboratory experiments Biol Rev 2009 84 91 111 10.1111/j.1469-185X.2008.00064.x 19046398

[B9] Minagawa M Wada E Stepwise enrichment of 15 N along food chains-further evidence and the relation between δ15N and animal age Geochim Cosmochim Ac 1984 48 1135 1140 10.1016/0016-7037(84)90204-7

[B10] Ben-David M Schell DM Mixing models in analyses of diet using multiple stable isotopes: a response Oecologia 2001 127 180 184 10.1007/s004420000570 24577647

[B11] Vanderklift MA Ponsard S Sources of variation in consumer diet δ15N enrichment: a meta-analysis Oecologia 2003 136 169 182 10.1007/s00442-003-1270-z 12802678

[B12] Robbins CT Felicetti LA Sponheimer M The effect of dietary protein quality on nitrogen isotope discrimination in mammals and birds Oecologia 2005 144 534 540 10.1007/s00442-005-0021-8 15800751

[B13] Caut S Angulo E Courchamp F Variation in discrimination factors (δ15N and δ13C): the effect of diet isotopic values and applications for diet reconstruction J Appl Ecol 2009 46 443 453 10.1111/j.1365-2664.2009.01620.x

[B14] Caut S Angulo E Courchamp F Caution on isotopic model use for analyses of consumer diet Can J Zool 2008 86 438 445 10.1139/Z08-012

[B15] Federation of Animal Science Societies Guide for the care and use of agricultural animals in research and teaching 1999 2 Champaign, IL

[B16] Thiex NJ Manson H Anderson S Persson JA Determination of crude protein in animal feed, forage, grain, and oilseeds by using block digestion with a copper catalyst and steam distillation into boric acid: Collaborative study J AOAC Int 2002 85 309 317 11990013

[B17] Association of official analytical chemists [AOAC] Official methods of analysis 2000 17 Association of Official Analytical Chemists, Arlington (VA)

[B18] Association of official analytical chemists [AOAC] Official methods of analysis 1995 16 Association of Official Analytical Chemists, Arlington (VA)

[B19] Jim S Jones V Ambrose SH Evershed RP Quantifying dietary macronutrient sources of carbon for bone collagen biosynthesis using natural abundance stable carbon isotope analysis Brit J Nutr 2006 95 1055 1062 10.1079/BJN20051685 16768826

[B20] Deniro MJ Epstein S Mechanism of carbon isotope fractionation associated with lipid-synthesis Science 1977 197 261 263 10.1126/science.327543 327543

[B21] Deniro MJ Epstein S Influence of diet on the distribution of nitrogen isotopes in animals Geochim Cosmochim Ac 1981 45 341 351 10.1016/0016-7037(81)90244-1

[B22] Phillips DL Gregg JW Source partitioning using stable isotopes: coping with too many sources Oecologia 2003 136 261 269 10.1007/s00442-003-1218-3 12759813

[B23] Tieszen LL Boutton TW Tesdahl KG Slade NA Fractionation and turnover of stable carbon isotopes in animal-tissues implications for delta-C-13 analysis of diet Oecologia 1983 57 32 37 10.1007/BF00379558 28310153

[B24] De Smet S Balcaen A Claeys E Boeckx P Van Cleemput O Stable carbon isotope analysis of different tissues of beef animals in relation to their diet Rapid Commun Mass Sp 2004 18 1227 1232 10.1002/rcm.1471 15164353

[B25] Reich KJ Bjorndal KA del Rio CM Effects of growth and tissue type on the kinetics of C-13 and N-15 incorporation in a rapidly growing ectotherm Oecologia 2008 155 651 663 10.1007/s00442-007-0949-y 18188602

[B26] MacAvoy SE Arneson LS Bassett E Correlation of metabolism with tissue carbon and nitrogen turnover rate in small mammals Oecologia 2006 150 190 201 10.1007/s00442-006-0522-0 16967272

[B27] MacAvoy SE Macko SA Arneson LS Growth versus metabolic tissue replacement in mouse tissues determined by stable carbon and nitrogen isotope analysis Can J Zool 2005 83 631 641 10.1139/z05-038

[B28] Phillips DL Eldridge PM Estimating the timing of diet shifts using stable isotopes Oecologia 2006 147 195 203 10.1007/s00442-005-0292-0 16341714

[B29] Moreno-Rojas JM Tulli F Messina M Tibaldi E Guillou C Stable isotope ratio analysis as a tool to discriminate between rainbow trout (O. mykiss) fed diets based on plant or fish-meal proteins Rapid Commun Mass Sp 2008 22 3706 3710 10.1002/rcm.3775 18973201

[B30] Arneson LS MacAvoy SE Carbon, nitrogen, and sulfur diet-tissue discrimination in mouse tissues Can J Zool 2006 84 989 995

[B31] Bauchinger U McWilliams S Carbon turnover in tissues of a passerine bird: allometry, isotopic clocks, and phenotypic flexibility in organ size Physiol Biochem Zool 2009 82 787 797 10.1086/605548 19785542

